# The WHO Disease Outbreak News during the Covid-19 pandemic

**DOI:** 10.1371/journal.pgph.0004025

**Published:** 2025-01-27

**Authors:** Ciara M. Weets, Colin J. Carlson, Hailey Robertson, Kate Toole, Lauren McGivern, Ellie Graeden, Rebecca Katz

**Affiliations:** 1 Center for Global Health Science and Security, Georgetown University Medical Center, Washington, DC, United States of America; 2 Department of Epidemiology of Microbial Diseases, Yale University School of Public Health, New Haven, Connecticut, United States of America; Mahidol University, THAILAND

## Abstract

During the Covid-19 pandemic, the World Health Organization (WHO) was an important public source of information – not only about the pandemic, but also thousands of other potential health emergencies. Here, we examine the 242 reports published in the WHO Disease Outbreak News (DON) during the first four years of the Covid-19 pandemic (2020 to 2023), and document the diseases and regions that were reported. We find that multinational epidemics of diseases like Ebola virus and MERS-CoV continue to dominate the DON. However, recent years have also seen more reports of climate-sensitive infectious diseases, as well as a state shift in influenza outbreak reporting in both China and the rest of the world. Surprisingly, the DON was only minimally used to document the Covid-19 pandemic and the global clade II mpox epidemic, almost exclusively before the declaration of a public health emergency of international concern. Notably, inconsistent reporting related to Covid-19 variants of concern speaks to the ongoing evolution of the DON as a resource, and potentially, to its complicated relationship with international travel and trade restrictions. We suggest that researchers should continue to exercise caution when treating the DON as a global record of outbreak history, but that the DON is a compelling record of the WHO itself, including the process it uses to assess outbreak risk.

## Introduction

Timely notification of infectious disease outbreaks is one of the primary determinants of rapid response, and as such, is consistently one of the strongest predictors of outbreak containment and severity [1]. For outbreaks of potentially high-consequence pathogens, including those of unknown origin or with known epidemic potential, the international notification process becomes particularly relevant. Under Article 6 of the International Health Regulations (IHR) (2005), when outbreaks meet certain criteria (outlined in Annex 2), Member States are required to notify the World Health Organization (WHO); under Article 9, the WHO is also empowered to collect information from other sources such as news outlets or voluntary reports from Member States. The WHO then synthesizes and verifies this information, and shares updates with Member States and – in some cases – with the public.

In these cases, the most frequently used written outlet (and the only official public record of outbreak history curated by the WHO) is the Disease Outbreak News (DON), an online resource that has been curated since 1996 and captures thousands of outbreak reports from around the world. Reports in the DON usually capture the outbreak location, the causative agent (if known) or symptomology, and other critical information, such as case counts, details on response efforts, or WHO guidance. Between January 1996 and December 2019, a total of 2,789 reports were published in the DON, capturing developments in major epidemics like the 2014 West African Ebola epidemic, unusual events detected by syndromic surveillance, and everything in-between. The DON is generally understood to be an incomplete record of infectious disease outbreaks: the diseases and countries that are represented have shifted through time, reflecting not just the global history of outbreaks, but also the priorities of the WHO and the concerns of Member States. Over the last two decades, the DON has become dominated by major viral epidemics (such as MERS-CoV or Ebola virus outbreaks), while reports related to infectious disease of poverty (e.g., cholera) or isolated cases of novel pathogens have become more sparse [2].

Here, we revisit these trends, and explore how the WHO has used the DONduring the Covid-19 pandemic. We develop an incremental update to an existing dataset capturing the focus of these reports [2], with 242 new records from between 2020 and 2023. We explore how these reports documented the Covid-19 pandemic itself, as well as other major epidemics and smaller outbreaks during the same time frame.

## Methods

### The Disease Outbreak News

Previously, we developed a standardized dataset capturing basic information related to, and the topics covered in, the WHO Disease Outbreak News between 1996 and 2019. Here, we expand that database during the four years of the Covid-19 public health emergency of international concern (PHEIC) (2020–2023), using an updated and simplified data collection procedure.

We reviewed all 242 reports published between January 1, 2020 and December 31, 2023 on the WHO website (www.who.int/emergencies/disease-outbreak-news). For each report, we captured the unique URL of the post (*Link*), the report title (*Headline*), and publication date (*ReportDate*). We captured outbreak geography at the country level (*Country,* as well as 3-letter ISO codes: *ISO*) or above. In reports on multi-country outbreaks within fewer than ten named countries, each report-disease-country combination was entered as a separate row. In some cases, countries were left unspecified and ISO codes were left blank if reports had an explicitly regional (e.g., “Americas”) or global focus. In keeping with the original data collection effort, we recorded a standardized set of disease names based on the language most commonly used in the reports (rather than any external taxonomy such as the International Classification of Diseases). All outbreaks are classified to the first level (*DiseaseLevel1*; e.g., “Polio”), and occasionally pathogen strains or similar granular data are also recorded (*DiseaseLevel2*; e.g., cVDPV2 or WPV:WPV1). Events of unknown etiology captured through syndromic surveillance are systematically categorized within one of seven “Syndromic” disease types (cardiovascular, diarrheal, gastrointestinal, haemorrhagic, hepatological, neurological, and respiratory).

### Case data

Covid-19 case data used in Fig 2 was taken from the publicly-available COVID-19 Data Repository published by the Center for Systems Science and Engineering (CSSE) at Johns Hopkins University [3]. These data are complete through March 10, 2023, when this project stopped collecting new data. Mpox case data was taken from the publicly-available Global.health repository [4]. Data were accessed on January 25, 2024, and are complete through December 13, 2023.

## Results

**Fig 1:**
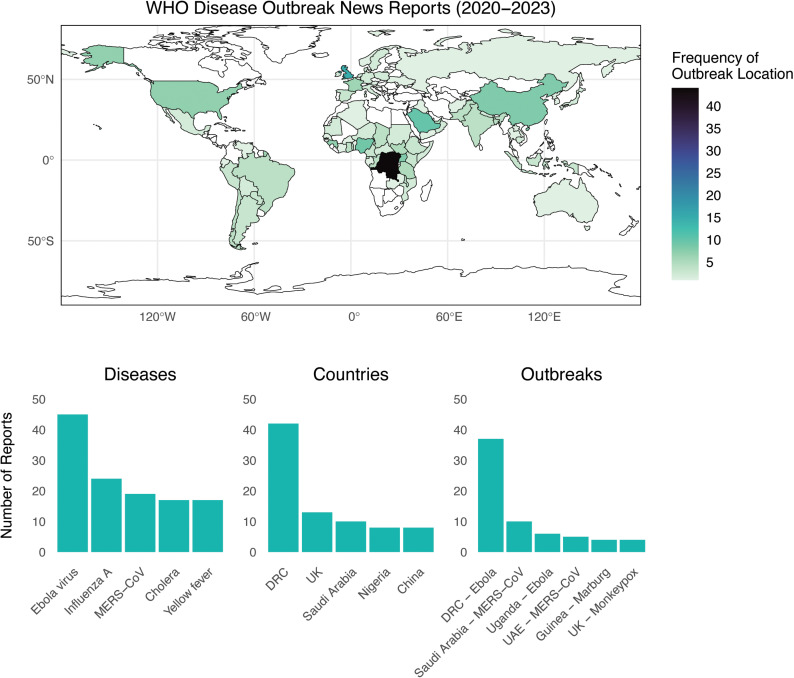
The global distribution of outbreak reporting and subject matter captured in the DON during the first four years of the Covid-19 pandemic. Figure created in R, utilizing the rnaturalearth package, which draws upon a public domain map dataset [8].

During the three-and-a-half-year period in which the Covid-19 PHEIC declaration was in place (2020 to 2023), the World Health Organization published a total of 242 reports in the Disease Outbreak News, describing outbreaks of 42 diseases in 89 countries (Fig 1).

### Covid-19 in the DON

Reports related to the Covid-19 pandemic accounted for a small proportion (3%) of total outbreak reporting between 2020 and 2023: only 9 reports were published related to Covid-19, all from 2020. An initial cluster of five reports were published between January 5, 2020 and January 17, 2020. The first report, on January 5 – six days after the first ProMed-mail report on December 30, 2019 – noted a pneumonia outbreak of unknown etiology in China, which the WHO China Country Office had become aware of on December 31, 2019. Notably, this report included some of the first public information related to connections between initial case clusters and Huanan Seafood Wholesale Market. Subsequent reports provided a detailed update on the situation in China, including identification of the novel coronavirus (January 12); and updates on imported cases in Thailand, Japan, and South Korea (January 14, 16, 17, and 21). On January 21, the WHO also began posting daily situation reports on COVID-19 (instead of using the DON); this continued until August 16, 2020, when reports transitioned to a weekly basis.

**Fig 2:**
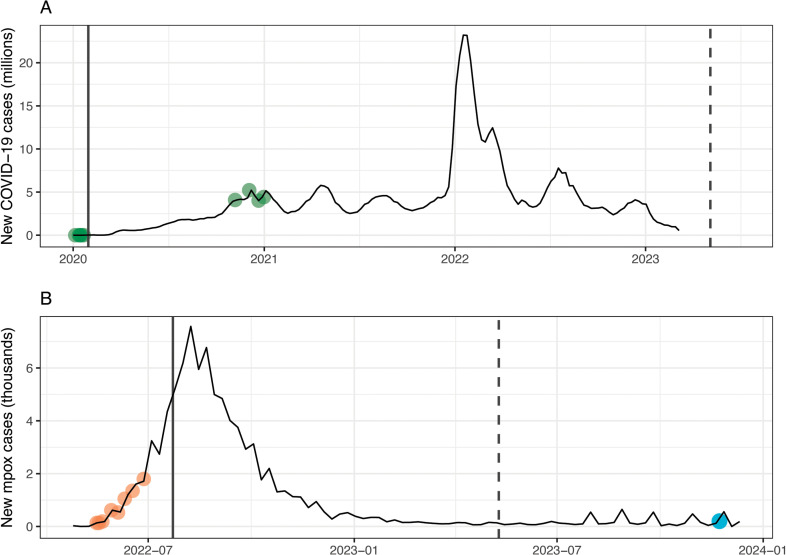
Weekly case counts and the timing of DON reports related to (A) the Covid-19 pandemic, and (B) the global clade II mpox epidemic. Transparent orange circles indicate points where a report about the outbreak was published in the DON. Solid lines indicate the date of PHEIC declaration, and dashed lines indicate the end of the declaration.

Covid-19 did not reappear in the Disease Outbreak News again until November 6, 2020, when a report described a novel variant that had emerged on mink farms in Denmark, which had been transmitted from mink to humans, and had acquired mutations with an unknown level of associated risk; an update on this situation was published on December 3, 2020. Another novel variant of concern that had begun to spread rapidly in the United Kingdom was noted on December 12, 2020 (“SARS-CoV-2 VUI 202012/01,” now referred to as the Alpha variant). A final report was published on December 31, 2020, providing a global update of the first year of the pandemic, including references to the variants of concern that had been recently detected in Denmark, the United Kingdom, and South Africa (“501Y.V2,” now Beta), and the ongoing challenge of genomic surveillance. At the time of writing, this is the last time that the Disease Outbreak News was used to provide updates on Covid-19. Notably, no reports were published in response to the Omicron variant, which was significantly more transmissible than all other previous variants, and drove a global spike in cases, hospitalizations, and deaths (Fig 2).

### Non-pandemic outbreaks in the DON

A total of 42 different diseases, including SARS-CoV-2, were reported in Disease Outbreak News between 2020 and 2023. During this period, the DON captured several multinational epidemics and situations, including the global clade II mpox epidemic, multiple outbreaks of haemorrhagic fevers, and the global avian influenza (A/H5N1) panzootic.

Viral haemorrhagic fevers dominated the Disease Outbreak News between 2020 and 2023. Whereas Ebola virus was the third-most reported disease between 1996 and 2019 (Carlson et al. 2023), it was the most reported disease between 2020 and 2023, accounting for 17% of all reports (45 of 258). Most of these reports (64%) were related to the 2018 outbreak originating in the Democratic Republic of the Congo, which was declared a PHEIC on July 17, 2019 (nearly a year into the epidemic); the declaration was lifted on June 26, 2020. Even after this outbreak ended, Ebola virus remained the most reported disease in 2021 and 2022, and Marburg virus was the most reported disease in 2023. Lassa fever also appeared in 6 reports, including a February 9, 2022 report of an imported case in the United Kingdom.

The global epidemic of clade II mpox was the first PHEIC declared by the World Health Organization since the start of Covid-19, and was concurrent with the Covid-19 emergency, with WHO deciding to lift the two emergency declarations a week apart. Like the Covid-19 pandemic, this mpox epidemic only featured minorly in the DON. A total of 14 reports related to mpox were published by the end of 2023, with all but one preceding the emergency declaration on July 23, 2022 (Fig 2B). The first report, published on October 1, 2020, described an outbreak of over 4,000 cases in the Democratic Republic of the Congo; while mpox is endemic in central and west Africa, the outbreak was characterized by an abnormally high case-fatality rate (4.2%) in children under five. In 2021, a total of four reports described imported cases of mpox that had been detected in the United States and United Kingdom, but these are believed to have been contained. Between May 16, 2022 and June 27, 2022, a series of eight reports described the emergence of an epidemic situation in high-income countries, primarily experienced by communities of men who have sex with men. On July 6, 2022, the World Health Organization transitioned to weekly situation reports instead of updates in the DON. A final report was published on November 27, 2023, drawing attention to the rapidly expanding outbreak of clade I mpox in the Democratic Republic of the Congo (an epidemic that was eventually declared a PHEIC on August 14, 2024).

Reporting patterns related to influenza shifted notably during the four-year period. Historically, influenza A has been the most reported pathogen in the Disease Outbreak News, with reports from China being the most common country-disease pair. During the Covid-19 pandemic, public reporting from the World Health Organization related to health emergencies in China appears to have declined; out of 8 total reports specific to China, just 3 were related to influenza A cases. However, influenza A remained the second-most reported pathogen, with a number of reports of spillover from countries around the world—particularly related to the ongoing avian influenza (A/H5N1) panzootic. A handful of novel subtypes were also reported in the DONs, such as H5N8 in Russia (2020), H10N3 in China (2021), and H3N8 in China (2022), suggesting that these countries continued to comply with their obligation to report human infections with novel influenza A subtypes under the International Health Regulations (2005). One explanation for reduced reporting might be that the Covid-19 pandemic has made it much harder to distinguish causes of influenza-like illness, especially based on syndromic surveillance alone.

Finally, during the Covid-19 pandemic, the DON documented a notable increase in outbreaks of climate-sensitive infectious diseases, especially vector-borne diseases such as yellow fever (25 reports) and dengue fever (14 reports), and water-borne diseases such as cholera (17 reports). These trends speak to the growing impacts of climate change on the global burden of disease, and to WHO’s growing recognition of these risks.

## Discussion

During the Covid-19 pandemic, the WHO was tasked with responding to an unprecedented number of concurrent health emergencies (as well as unprecedented levels of outbreak-related mis- and dis-information). The WHO has several means by which they are able to share information with both Member States and the public, including the Disease Outbreak News, the *Weekly Epidemiological Record,* situation reports, press releases, press conferences, social media posts, and non-public communications [5]. The decision to use any of these channels to share information – or equally, a decision not to do so – can prompt speculation about the risk assessment process and political pressures faced by the organization.

Over the last few years, the Health Emergencies Programme has made a concerted and public effort to improve transparency. During the pandemic, the WHO launched a new web portal for the Health Emergencies Programme, complete with a new search interface for the DON. The website also added language about the criteria used to decide which outbreaks are covered:

*Disease Outbreak News (DONs) are published relating to confirmed or potential public health events, of:*

- *Unknown cause with a significant or potential international health concern that may affect international travel or trade;*
- *A known cause which has demonstrated the ability to cause serious public health impact and spread internationally;*
- *High public concern which may lead to disruption of required public health interventions, or could disrupt international travel or trade.*

These criteria closely mirror the Annex 2 decision framework that States are required to use to assess potential public health emergencies of international concern under the IHR (2005). However, both Article 6 and 9 notifications from Member States are sometimes kept confidential per the wishes of the reporting State, and only a subset of these outbreaks are captured in the DON.

In February 2024 (since the conclusion of our study), the criteria for DON publication were clarified in significantly greater depth by a peer-reviewed article in *BMJ Global Health*, co-authored by members of the Health Emergencies Programme from WHO headquarters and every regional office [6]. This article shared the decision instrument used to trigger publication of DON reports (for the first time, to our knowledge), establishing that reports are published following Member State notifications if information on the outbreak is already public, or if there is a “need to disseminate authoritative and independent information as per Article 11 of IHR (2005).” The authors also note that other considerations include “a need for assistance from the international community” and “potential public interest.” The article also shared that States are made aware of outbreaks through the confidential IHR Event Information Site (EIS) system prior to any DON report, with only rare exceptions (e.g., when updates on an outbreak are already being regularly published), and that affected States are usually consulted when a DON report is being prepared.

The article by Lata *et al.* also provided important insights into the purpose that the DON is meant to serve, and how this has shaped the evolving content of the reports. Lata *et al.* state that “while DON reports are available to the public, the primary audiences are health professionals, subject matter experts, and the media.” The emphasis on providing these practitioner audiences with authoritative information is explicitly linked to the growing burden of mis- and dis-information that WHO faced during the pandemic:

*…the rapid global expansion of the internet and the emergence of digital and social media have transformed the way in which society interacts with information. Information on acute public health events is more available and widely disseminated than ever before, but such information is not always verified or accurate. Furthermore, disinformation and misinformation are actively occurring, including during and in response to public health events. As an important counterbalance, DON reports aim to provide accurate, timely and authoritative information.*

This suggests that specific mis-/dis-information may be considered under the stated criteria for report publication (i.e., a need to provide independent, authoritative information), and helps explain the growing detail of the reports. However, we note that this role for the DON is perhaps expanding or still being explored, given that the DON were only minimally used to address these issues for Covid-19 or mpox. Lata *et al.* also share that the DON format and process is periodically reviewed in an expert consultation, most recently in 2019. In the currently used format, reports are described as “highly standardised,” with seven sections used across all reports currently: situation at a glance; description of the situation; epidemiology of the disease; public health response; WHO risk assessment; WHO advice; and further information. They also observe that visualizations are used more commonly now, although “timely dissemination of DON reports takes precedence over the inclusion of new visualisations.”

The publication of this documentation is a significant advance in the level of transparency with which the DON is handled. This has clarified WHO’s perspective on the purpose served by the DON relative to their obligations under its Constitution and the IHR (2005), and confirms some long-standing suspicions, such as the deliberate use of the DON as a tool to draw attention to unfolding humanitarian crises [2]. It also underscores the unique value proposition of the DONs compared to, say, the all-purpose outbreak reporting in sources like ProMed-mail and HealthMap: they represent a first-order, qualitative risk assessment by the WHO related to which outbreaks have become, or are likely to become, emergencies.

As the WHO has become clearer about the purpose of the DON, the DON itself has become a more revealing record of the WHO’s internal process. Potentially high-consequence outbreaks that are subject to clear notification obligations under IHR Article 6 and Annex 2 – such as novel subtypes of influenza, viral haemorrhagic fever outbreaks, or poliomyelitis outbreaks – will likely continue to dominate the DON for the foreseeable future. The growing representation of climate-sensitive diseases similarly speaks to WHO’s recognition of their growing potential to cause disruptions to travel and trade in Africa and Latin America especially. The most interesting cases may be where WHO breaks from its own process: for example, the Alpha and Beta variants of Covid-19 were recorded in the DON, but the Omicron variant, which not only had a higher impact on human health, but also led to more significant international disruptions of travel and trade, was not. Decisions like these may simply be stochastic and may be reflective of high-consequence, limited-time frame decision-making, but they may also sometimes reflect the challenges faced by the WHO as global governance changes. For example, the Omicron variant led to an unprecedented breakdown of international norms around travel restrictions, which now poses an enduring problem in future outbreaks [7]. It remains to be seen how these new challenges will change the baseline process of information sharing during health emergencies—making the DON a more important resource than ever.
